# Cue labeling reduces cigarette craving and associated neural activity

**DOI:** 10.1038/s41386-025-02297-8

**Published:** 2025-12-17

**Authors:** Golnaz Tabibnia, Dara G. Ghahremani, Hilary A. Tindle, J. David Creswell, Cecilia Westbrook, Thomas E. Kraynak, Erica Julson, Edythe D. London

**Affiliations:** 1 Department of Psychology, University of California, Irvine, CA USA; 2 Department of Psychiatry and Biobehavioral Sciences, Semel Institute for Neuroscience and Human Behavior, University of California, Los Angeles, CA USA; 3 Department of Medicine, Vanderbilt University Medical Center, Nashville, TN USA; 4 Geriatric Research Education and Clinical Center for Healthy Longevity, Veterans Affairs Tennessee Valley Healthcare System, Nashville, TN USA; 5 Department of Psychology, Carnegie Mellon University, Pittsburgh, PA USA; 6 Department of Psychiatry, University of Pittsburgh, Pittsburgh, PA USA

**Keywords:** Translational research, Human behaviour

## Abstract

Cigarette craving, triggered by smoking-related cues, significantly contributes to resumption of smoking after cessation. This study investigated whether affect labeling, the cognitive technique of using words to regulate emotions, could be adapted to regulate cue-induced craving. Fifty adults who smoked cigarettes daily (24 women, ages 20–65) completed a novel Cue Labeling Task during fMRI. In this task, participants viewed cigarette-cue images under three conditions: cue matching (craving elicitation), cue labeling (experimental condition), and gender labeling (control). In a fourth condition, neutral matching (baseline), they matched neutral images with one another. Participants rated their cigarette craving in each condition. Relative to cue matching, cue labeling elicited lower craving (*p* < 0.05, Hedges’ *g* = −0.11) and lower activation in the precuneus (*Z* = −4.5, *p*_corrected_ < 0.001), a region associated with craving in prior research and in the present study (*β* = 0.43, *p* < 0.05). Age moderated these effects. In the older (but not younger) subsample (Johnson-Neyman age cutoff of 46.7 years), craving during cue labeling was lower than cue matching (*p* < 0.05, *g* = −0.29) and similar to neutral matching (*p* > 0.5). Greater age was also associated with lower precuneus activity during cue labeling versus cue matching (*Z* = −3.8, *p*_corrected_ < 0.001). Gender labeling did not significantly alter craving compared to cue matching (*p* > 0.05, *g* = −0.13); and, although it led to lower precuneus activity (*Z* = −5.5, *p*_corrected_ < 0.001), activity in this region was lower during cue labeling than gender labeling (*Z* = −3.9, *p*_corrected_ < 0.001). These findings suggest that cue labeling, a simple and scalable self-regulation strategy, may reduce cigarette craving and related neural activity, particularly among older midlife adults with nicotine addiction.

## Introduction

Each year, about half of U.S. adults who smoke cigarettes attempt to quit, yet fewer than 10% sustain abstinence for six months or longer [1, 2]. Cigarette craving, often triggered or intensified by smoking-related cues, undermines quitting [3, 4]. In fact, exposure to nicotine cues poses a greater risk of relapse than nicotine dependence severity [3]. Although cognitive strategies can reduce cue-induced craving and associated neural activity [5–7], these strategies typically require training and cognitive resources [8–10], which may be compromised during nicotine withdrawal [11] or in vulnerable populations such as older adults [12]. The present study tested whether affect labeling, a simple cognitive strategy for reducing negative *affect*, could be adapted as a novel strategy for reducing *craving* among people who smoke.

Affect labeling involves putting words to emotional experience or to emotion-eliciting stimuli. In laboratory studies, choosing affective verbal labels for emotionally evocative images (versus matching evocative images to one another) reduces self-reported distress, physiological arousal, and activity in the amygdala, a brain region central to fear and arousal [13–16]. Choosing non-affective labels (e.g., gender labels) does not attenuate amygdala activation, indicating that the effect is specific to affect labeling rather than a general consequence of labeling or distraction [14, 15]. Supporting its relevance in naturalistic settings, an analysis of roughly 67,000 Twitter posts showed that emotional intensity in messages declines rapidly after affect labeling statements (e.g., “I feel awful”) [17].

The benefits of affective labeling can extend beyond immediate relief. In laboratory analogs of exposure therapy, repeated pairings of aversive images with affective labels produced greater reductions in autonomic reactivity to those images one week later compared to repeated exposure to the images alone [18]. In clinical trials, affective labeling improved exposure therapy outcomes in individuals with spider phobia [19] and public speaking anxiety [20]. In fact, affect labeling has been recommended as a strategy to optimize exposure-based interventions [21].

These findings support the long-standing view that putting feelings into words alters emotional experience [22], a principle widely applied in psychotherapy. Building on this framework, the present study asked whether labeling craving-eliciting cues can reduce craving, just as labeling emotion-eliciting cues reduces affective responses. Prior work shows that affect labeling attenuates positive affect in response to pleasant cues [13] and decreases amygdala activation to emotional faces in individuals with substance use disorder [23]. Yet, the potential impact of labeling on *craving* remains unknown.

Labeling may plausibly reduce craving, given the psychological and neural overlap between craving and negative affect. Self-reported craving and negative affect are often correlated [24–27], and both contribute to substance use and relapse [25, 28, 29]. Regulation of emotion and craving recruits overlapping regions of the prefrontal cortex in tobacco use [30] and alcohol use [31] disorders. Moreover, cigarette craving and negative affective states appear to share a common neural substrate within the default mode network (DMN), specifically in the medial parietal cortex [27]. The medial parietal cortex―including the posterior cingulate cortex (PCC) and precuneus―is a DMN hub, implicated in self-referential thought [32, 33], including rumination [34, 35] and reflecting on one’s current experience [36]. Heightened activity in this region has been observed during early abstinence from smoking [37] and in response to smoking cues [38–41], and it has been linked to slips in abstinence [42]. Notably, affect labeling reduces activity in this region in healthy adults [14, 43], as does mindfulness training [7].

Individual differences that may moderate the effects of labeling are not well understood. A large-scale analysis of Twitter posts identified potential gender differences in emotional dynamics after labeling [17], and another study found neural response to affect labeling in social phobia to vary depending on comorbid depression [44]. Research on cognitive reappraisal, a related emotion-regulation strategy, suggests that its effects can also vary by sex/gender [45, 46] and mental health status [47], as well as by age [48]. For example, some studies report that cognitive reappraisal is less effective in older than younger adults [49, 50]. Further, among older adults, greater use of cognitive reappraisal has been associated with lower levels of internalizing symptoms in women but not men [51]. Thus, age, sex/gender, and clinical symptoms may interact in complex ways to influence the impact of cognitive self-regulation on affect-related outcomes, and perhaps also craving.

Given evidence that negative affect and craving share psychological and neural substrates, particularly within the medial parietal cortex [26, 27, 52, 53], and that affect labeling reduces both negative affect and activity in this region [14, 43], we hypothesized that labeling cigarette cues would reduce both craving and activity in this and other craving-related regions, including the striatum and anterior cingulate cortex (ACC) [40, 54]. To investigate this possibility, we developed the Cue Labeling Task and tested its effect on regulating cue-induced craving during functional magnetic resonance imaging (fMRI) in adults who smoked cigarettes daily. In this task, participants viewed either neutral target images (e.g., a cup) or cigarette-cue target images (e.g., a pack of cigarettes), and they selected one of two words (labeling condition) or one of two images (matching condition) that better corresponded to the target image. In the cue labeling condition, participants were asked to label features of the cues (e.g., “smoke”) rather than their own subjective state (e.g., “craving”), because the former may be more effective in regulating reactions to evocative stimuli [16].

We tested the following pre-registered hypotheses (osf.io/4dfsv): 1) labeling cigarette cues with cigarette-related words (e.g., “puff” or “smoke”), compared to matching cigarette cues, will decrease self-reported craving and activation in medial parietal cortex and other craving-related regions, including the cingulate cortex and striatum; and 2) labeling cigarette cues with non-cigarette words (e.g., “man” or “woman”) will not reduce craving or activation in these regions. We also explored whether age, sex, and nicotine dependence moderate the effects of labeling on craving-related outcomes.

## Materials and methods

### Participants

Sixty-two treatment-seeking adults who smoked cigarettes daily were recruited as part of the Healthier Brains in Treating Smoking trial (NCT00581464, P.I. Tindle). Eligibility criteria included smoking at least 10 cigarettes per day, reporting a strong desire to quit, willingness to participate in smoking-cessation treatment, and age 18-65 years. Exclusion criteria included pregnancy, concurrent substance use (verified by urinalysis), use of medications affecting the nervous system (e.g., analgesic or psychotropic drugs), history of brain injury, cognitive impairment, and untreated psychiatric illness. All procedures were approved by the institutional review boards at the University of Pittsburgh and Carnegie Mellon University.

### Procedures

During a baseline visit, prior to the fMRI visit, participants provided written informed consent and completed questionnaires assessing smoking history, demographics, nicotine dependence (Fagerström Test for Nicotine Dependence, FTND) [55], and depressive symptoms (Beck Depression Inventory II, BDI) [56] (see Supplementary Methods). Pack-years of smoking exposure was computed as cigarettes per day × years of smoking/20. Scanning was conducted prior to treatment. Participants were instructed to abstain from smoking for 12 h prior to scanning to assess craving-regulation during early abstinence, when self-regulation is particularly critical for preventing lapse and maintaining abstinence. Immediately before scanning, exhaled carbon monoxide was measured, and urine toxicology was performed to screen for cocaine, tetrahydrocannabinol, methamphetamine, and opioids. Participants who tested positive for any substance were rescheduled. During the scan, participants completed the cue labeling task, as well as other tasks described elsewhere [30, 57].

### Cue labeling task

**Fig. 1:**
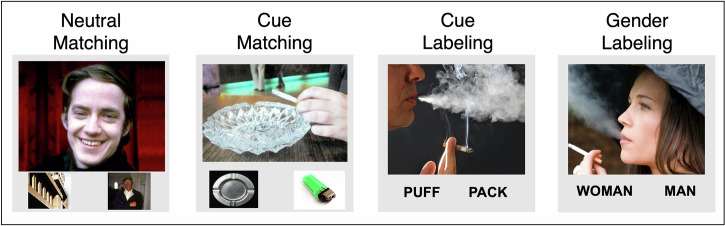
Sample screens from each of the four conditions. In neutral matching, participants selected one of two small images of ordinary objects, buildings, or people (i.e., neutral images), that matched a larger neutral image. In cue matching, participants selected one of two cigarette-related images that matched a larger cigarette-cue image. In cue labeling, participants selected one of two cigarette-related words to match a cigarette-cue image. In gender labeling, they selected one of two non-cigarette words that matched the gender of the person in the cigarette-cue image. (Images were obtained from the International Affective Picture System (IAPS) [58], the International Smoking Image Series (ISIS) [59], or purchased online at istockphoto.com.).

The task comprised four experimental conditions: neutral matching, cue matching, cue labeling, and gender labeling (Fig. 1). In all conditions, participants viewed a large target image and selected one of two corresponding options beneath it. In neutral matching, participants selected one of two small images of ordinary objects, buildings, or people (i.e., neutral images), that corresponded to the target neutral image. In cue matching, participants selected one of two cigarette-related images that corresponded to the target cigarette-cue image. In cue labeling, participants selected one of two cigarette-related words (e.g., “PUFF” or “PACK”, “SMOKE” or “MOUTH”) that corresponded to a cigarette-cue target image. In gender labeling, they selected one of two non-cigarette words (e.g., “MAN” or “WOMAN”, “BOTH” or “NEITHER”) that corresponded to the gender(s) of the person(s) in the target cigarette-cue image. Condition order was counterbalanced across participants, with each condition appearing equally often in each of the eight block positions in each run.

The neutral images were obtained from the International Affective Picture System (IAPS) [58]. The smoking-related images were obtained from the International Smoking Image Series (ISIS) [59] or purchased online at istockphoto.com. Stimuli were counterbalanced across conditions for ratings of arousal and valence (neutral images) and for craving ratings (cigarette images) (see Supplementary Methods).

There were 24 trials per condition, presented in four 6-trial blocks. Each block of trials began with a 2.5-s instruction screen (“Match” or “Label”). Each trial comprised a 4-s period during which participants viewed the smoking or neutral image and selected one of the two response options. Each trial was followed by a fixation cross of jittered duration (average 1 s). At the end of each block, participants rated their craving on a 5-point scale, followed by a 5-s rest before the start of the next block (Fig. S1). Participants indicated their responses using a response unit with a button for each finger (Psychology Software Tools, Pittsburgh, PA, USA).

### MRI data acquisition

Brain images were acquired on a Siemens Allegra 3-T scanner with a single-channel head coil. High-resolution T1-weighted magnetization prepared rapid gradient echo images (isovoxel 0.8mm^3^, FOV = 205 mm, matrix = 256 × 256, TR = 1.63 s, TE = 2.48 ms, flip-angle = 8, 224 sagittal slices) were acquired for spatial registration of the fMRI data, which were acquired using an echo-planar pulse sequence (isovoxel 3.2 mm^3^, FOV = 205 mm, matrix-size = 64 × 64, TR = 2 s, TE = 28 ms, flip-angle = 79, slice-thickness = 3.2 mm, no slice gap, 34 oblique-axial slices). Two Cue Labeling Task runs were acquired, each lasting 5:40 minutes and comprising 162 volumes.

### Self-report analyses

Linear mixed models (LMMs) were used to examine the effects of condition on self-reported craving. Specifically, the contrasts of interest were: 1) cue matching vs. neutral matching, as a manipulation check that the smoking cues elicited craving (i.e., a measure of cue-elicited craving); 2) cue matching vs. cue labeling, to examine the reduction in cue-induced craving due to labeling; and 3) cue matching vs. gender labeling, to test the effect of gender labeling, hence assessing the specificity of the cue labeling effect.

We used R 4.2.2 [60] and the *lme4* library [61] to test these LMMs. As fixed effects, we entered condition, age, sex, and FTND score (without interaction terms) into the model. As random effects, we included an intercept for “subjects” to model baseline differences across individuals. Interaction terms for sex, age, and FTND were included in all moderation analyses.

### fMRI analyses

The neuroimaging data were organized following the Brain Imaging Data Structure (BIDS) format [62] and preprocessed using fMRIPrep 23.1.4 [63]. Functional images were corrected for slice timing and motion, co-registered to the T1-weighted image, and normalized to the Montreal Neurological Institute (MNI) template (see Supplementary Methods). Preprocessed images were analyzed with the FSL 5.0.9 toolbox (FMRIB Analysis Group, Oxford, UK). After high-pass temporal filtering (100 s) and spatial smoothing (4-mm FWHM), time-series analyses were conducted using FSL’s FMRIB Improved Linear Model with local auto-correlation correction. Each 30-s task block, instruction screen, and craving-rating period was modeled as a custom waveform, convolved with a canonical hemodynamic response function (double-χ) with a width equaling the stimulus length and its temporal derivative.

A priori linear contrasts (identical to those used in the self-report analyses) were combined across the two runs using fixed-effects linear models and analyzed at the group level using FSL mixed-effects analysis (FLAME), with hierarchical linear modeling correcting for variance heterogeneity and non-sphericity. Age, sex, FTND score, and motion estimates (mean framewise displacement, mFD) [64] were modeled as covariates.

For exploratory brain-craving associations, parameter estimates during cue labeling were extracted from the precuneus cluster identified in the contrast of cue matching versus cue labeling, and they were regressed against self-reported craving during cue labeling. Linear regression models were used, with age, sex, FTND, and mFD as covariates.

To examine functional connectivity during cue labeling, connectivity analyses (Psychophysiological Interactions [65]) were conducted with 4 regressors in the first-level model: 1) cue labeling events, 2) seed time-courses, 3) connectivity term (product of regressors 1 and 2), and 4) all other events. Time-courses during cue labeling were extracted from a precuneus seed that was functionally-defined in the group contrast of cue matching versus cue labeling.

Cluster-based correction for tests on multiple voxels was used at a voxel-height threshold of *p* < 0.001 (*Z* > 3.1) and a cluster-size threshold of *p* < 0.05 [66]. For exploratory and *post hoc* analyses, a cluster-corrected voxel-height threshold of *p* < 0.01 (*Z *> 2.3) was used. Reported coordinates correspond to the peak voxel within each cluster or subcluster in MNI space.

## Results

### Data exclusion

Data from eight participants were excluded due to non-compliance with study protocol (e.g., falling asleep during scanning). Eight additional participants were excluded from self-reported craving analyses due to technical issues with the button response unit, resulting in a sample of *N* = 46 for craving analyses. These participants were retained in the neuroimaging analyses, as response unit issues are unlikely to affect the hypothesized neural responses; excluding these eight participants from fMRI analyses did not affect the main findings (Supplementary Table S1). Neuroimaging data from four other participants were excluded due to excessive head motion (mFD > 0.6 mm), resulting in an fMRI sample of *N* = 50.

### Participant characteristics

Table 1 summarizes demographic and clinical characteristics of the fMRI (*N* = 50) and self-report (N = 46) samples. In both samples, approximately half identified as female, identified as Caucasian–American, and reported annual household incomes below $20,000. Mean BDI scores were below the clinical threshold for depression (cutoff = 13). Mean FTND scores indicated moderate to high nicotine dependence in both the fMRI (mean (*M*) = 5.0, standard deviation (SD) = 2.2) and self-report (*M* = 4.8, SD = 2.3) samples. Levels of expired CO were generally consistent with overnight smoking abstinence, although 9 of 54 participants had CO > 10 ppm on the scan day. The two samples did not differ in any of the demographic or clinical variables (all *p*’s > 0.5).

**Table 1:** Participant Characteristics.

	fMRI (*N* = 50)	Craving (*N *= 46)
Age	45.9 (10.8)	45.4 (10.5)
Female	48%	52%
Race		
African–American	42%	48%
Caucasian–American	54%	50%
Other	4%	2%
Annual Household Income		
<$20,000	54%	52%
$20,000–50,000	20%	20%
$50,000–75,000	22%	24%
>$75,000	4%	4%
Beck Depression Inventory II		
Score	7.0 (5.4)	7.5 (6.5)
Scored ≤13	86%	83%
Smoking status		
Nicotine dependence (FTND)	5.0 (2.2)	4.8 (2.3)
Cigarettes per day	17.3 (6.1)	16.9 (6.8)
Pack-years	23.1 (13.2)	23.3 (14.0)
Baseline CO level (parts per million)	15.7 (8.6)	15.6 (7.3)
fMRI CO level (parts per million)	5.7 (4.3)	5.0 (3.8)

Notes. Results are presented as mean (standard deviation) or as a percentage. Age ranged from 20 to 65 in the fMRI sample and from 20 to 63 in the Craving sample.

“fMRI sample” includes all participants with fMRI data, including 8 participants who were excluded from self-reported craving analyses. “Craving sample” includes all participants with self-reported craving data, including 4 participants who were excluded from fMRI analyses (see Data Exclusion).

*CO* carbon monoxide, *FTND* Fagerstrōm Test for Nicotine Dependence.

### Main effects on craving

**Fig. 2:**
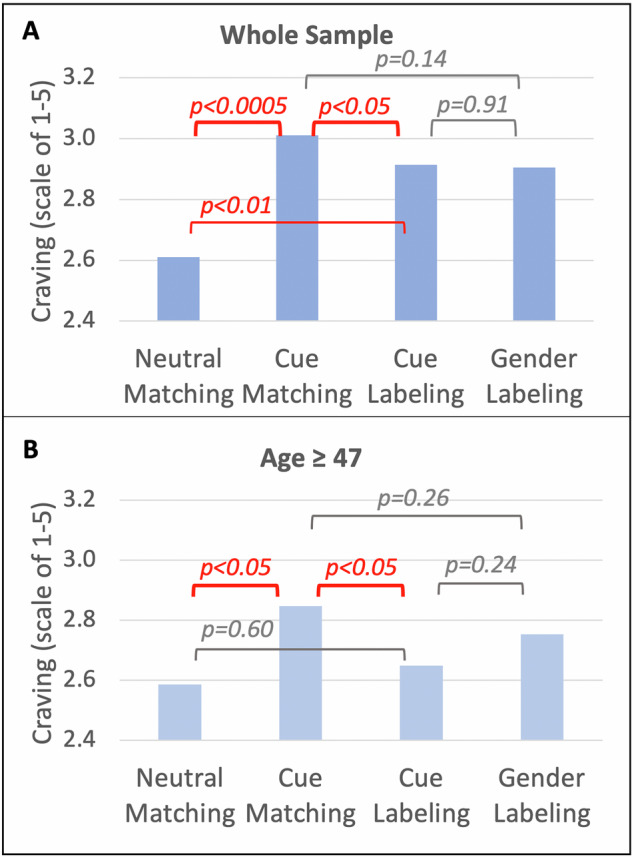
The effect of condition on self-reported craving. The effect is shown in the whole sample (**A**; *N* = 46) and in the older subsample (**B**; *N* = 23).

As a manipulation check, we first confirmed that smoking cues elicited craving (*F*_(1, 45)_ = 17.2, *p* = 0.0002; Hedges’ *g* = 0.46), as indicated by greater self-reported craving during cue matching (M = 3.01, SD = 0.88) than neutral matching (*M* = 2.61, SD = 0.86; Fig. 2A). Importantly, self-reported craving was lower during cue labeling (*M* = 2.91, SD = 0.96) than cue matching (*F*_(1, 45)_ = 4.1, *p* = 0.049; Hedges’ *g* = -0.11). In contrast, gender labeling (*M* = 2.90, SD = 0.79) did not have a statistically significant effect on craving (*F*_(1, 45)_ = 2.28, *p* = 0.14; Hedges’ *g* = −0.13) relative to cue matching.

### Main effects on neural activation

**Fig. 3:**
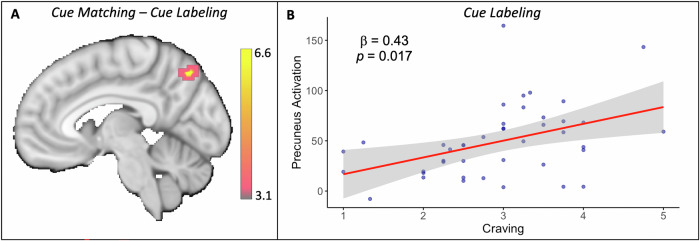
Precuneus response during cue labeling. **A** Precuneus activity is greater during cue matching than cue labeling. Color bar indicates Z-statistic values. **B** Precuneus activity during cue labeling is positively associated with self-reported craving during those trials (results remain significant after outlier removal; see Supplementary Materials and Fig. S7). Y-axis represents partial residuals of the fMRI contrast estimates during cue labeling. Data points are from the 42 participants with both craving and fMRI data.

Neural activity was lower during cue labeling than cue matching in a large posterior cluster that included the precuneus (subcluster peak: *x* = −4, *y *= −68, *z* = 50, *Z* = −4.8; Fig. 3A), along with other posterior clusters such as the fusiform (Table 2 and Supplementary Fig. S2). Neural activity was also lower during gender labeling than cue matching in the precuneus, along with multiple clusters in lateral occipital and temporal occipital cortices (Table 2 and Supplementary Fig. S3). However, a *post hoc* comparison indicated lower precuneus activity during cue labeling than gender labeling (Supplementary Table S2); no clusters in medial parietal cortex were more active in cue labeling than gender labeling. Relative to cue matching, both cue labeling and gender labeling increased activation in ventrolateral prefrontal cortex (Table 2).

**Table 2:** Brain activation during matching versus labeling of smoking cues, and during labeling versus matching of smoking cues.

Region^a^	Cluster size (voxels)	*Z* (max)^b^	*X*^c^	*Y*^c^	*Z*^c^
*Cue Matching–Cue Labeling*					
Lateral Occipital/Precuneus	470	6.64	34.8	-81.3	17.5
Lateral Occipital	162	4.79	−32.4	−87.7	20.7
Lingual Gyrus/T.O. Fusiform	114	5.26	28.4	−58.8	−4.9
T.O. Fusiform	50	4.94	−32.4	−55.6	−14.5
*Cue Matching–Gender Labeling*					
Precuneus	118	5.45	−13.2	−71.6	36.7
Lateral Occipital/Occipital Pole	110	5.27	34.8	−87.7	17.5
T.O. Fusiform	84	5.33	−26	−52.4	−14.5
T.O. Fusiform/Lingual Gyrus	77	5.7	25.2	−49.2	−14.5
Lateral Occipital/Occipital Pole	49	4.65	−32.4	−87.7	23.9
Lateral Occipital	36	4.4	50.8	−68.4	−8.1
Lateral Occipital / MTG	31	4.25	−45.3	−68.4	1.5
Lateral Occipital	30	4.52	22	−71.6	52.7
*Cue Labeling–Cue Matching*					
STG/MTG	88	4.93	−51.7	−36.4	1.5
IFG opercularis/Precentral Gyrus	36	5.09	−58.1	8.43	17.5
OFC/IFG triangularis	23	4.19	-42	27.7	-4.9
*Gender Labeling–Cue Matching*					
MTG/STG	282	5.3	−51.7	−39.6	1.5
IFG opercularis/triangularis	202	5.34	−54.9	18	23.9
IFG opercularis/triangularis	95	5.05	50.8	21.2	7.9
Angular Gyrus	84	4.6	57.2	−52.4	33.5

Whole-brain voxel-wise contrasts are presented. All results were cluster corrected at a voxel-height threshold of *p* < 0.001 (*Z* > 3.1) and a cluster-size threshold of *p* < 0.05.

*T.O.* Temporal Occipital, *STG* Superior Temporal Gyrus, *MTG* Middle Temporal Gyrus, *IFG* Inferior Frontal Gyrus, *OFC* Orbitofrontal Cortex.

^a^ Anatomical labels based on the Harvard-Oxford Structural Atlas.

^b^ *Z*-statistic of peak voxel.

^c^ MNI coordinates of peak voxel within cluster.

### Brain-craving associations

During cue labeling, precuneus activity was positively associated with self-reported craving (*β* = 0.43, *p* = 0.017; Fig. 3B). The association was specific to the precuneus; neural activity in other clusters in the contrast of cue matching versus cue labeling, such as the fusiform cortex (*β* = 0.26, *p* = 0.15), was not associated with self-reported craving during cue labeling. A *post hoc* mediation analysis indicated that the effect of labeling on craving was likely due to the impact of labeling on precuneus activity (see Supplementary Methods, Supplementary Results, and Fig. S4).

### Functional connectivity

Precuneus activity during cue labeling was positively associated with activity in several brain regions related to craving (Supplementary Table S3, Fig. S5), including the PCC, a cluster comprising the paracingulate gyrus and dorsal ACC, a cluster encompassing orbitofrontal cortex among other regions, and a cluster comprising dorsal thalamus and dorsal caudate.

### Individual differences

Age interacted with the effect of cue labeling (vs. cue matching) on craving (*F*_1,42_ = 5.28, *p* = 0.03), whereas sex (*F*_1,42_ = 0.72, *p* = 0.40) and FTND score (*F*_1,42_ = 0.01, *p* = 0.95) did not. As age increased, craving decreased during labeling.

Age also interacted with the effects of cue labeling on neural activity; as age increased, activation decreased during cue labeling (vs. cue matching) in precuneus (*x* = −4, *y* = −72, *z* = 30; *Z* = 3.8) and dorsal ACC (*x* = 12, *y* = 12, *z* = 27; *Z* = 4.1). FTND scores did not moderate labeling effects on brain activation. Sex moderated cue labeling effects, with lower activation in women than men during cue labeling in the frontal pole (*x* = 16, *y* = 47, z = −8; *Z* = 4.8), a white matter cluster that included part of the inferior frontal gyrus (*x* = −29, y = 5, *z* = 24; *Z* = 3.9), lateral occipital cortex (*x *= 45, *y* = −85, *z* = 14; *Z* = 3.8), and a white matter cluster near the supramarginal and angular gyri (*x* = 22, *y* = −43, *z* = 21; *Z* = 3.8).

To assess whether the moderating effects of age reflected greater exposure to smoking, we conducted *post hoc* analyses that included “pack-years by condition” as an interaction term in the LMM and pack-years as a covariate in the fMRI analysis. Pack-years did not interact with the effects of cue labeling on craving (*F*_1,42_ = 0.12, *p* = 0.74) or brain activity (*Z* > 2.3). See Supplementary Results for additional moderation analyses.

### *Post hoc* analyses in older participants

A Johnson-Neyman analysis [67, 68], conducted to identify the age interval within which cue labeling reduced craving, indicated that when age exceeds 46.7 years, the slope of cue labeling is *p* < 0.05. With the analyses limited to ages ≥47 years (*N* = 23; Fig. 2B), cue matching (*M* = 2.85, SD = 0.66) still elicited more craving than neutral matching (*M* = 2.59, SD = 0.70) (*F*_1,22_ = 6.68, *p* = 0.017; Hedges’ *g* = 0.38), and craving was lower during cue labeling (*M* = 2.65, SD = 0.70) than cue matching (*F*_1,22_ = 7.03, *p* = 0.015; Hedges’ *g* = −0.29). Notably, craving did not differ between the cue labeling and neutral matching conditions (*F*_1,22_ = 0.29, *p* = 0.60; Hedges’ *g* = 0.09). Gender labeling (M = 2.75, SD = 0.61) did not affect craving in this older group (*F*_1,22_ = 1.33, *p* = 0.26; Hedges’ *g* =−0.15). Activity in the precuneus was lower in both the older (*x* = 3, *y* = −75, z = 52; *Z* = −4.7) and the younger (*x* = −4, *y* = −72, *z* = 50; *Z* = -4.1) groups during cue labeling versus cue matching. See Supplementary Results, Tables S4–S5, and Fig. S6 for additional data on the older and younger subsamples.

## Discussion

Although linguistic labeling has long been proposed to modulate emotional responses, this is the first study to demonstrate efficacy of labeling in reducing craving. The main findings were: 1) cue labeling (compared to cue matching) reduced self-reported craving and activity in the precuneus, a brain region whose activity correlated positively with self-reported craving; 2) age moderated these effects of cue labeling, with older midlife participants showing greater reductions in both craving and precuneus activity; and 3) cue labeling led to lower precuneus activity than the control condition (gender labeling). Thus, consistent with our hypotheses, cue labeling was effective in reducing craving and its neural correlate; and unlike gender labeling, cue labeling significantly reduced craving, and it led to lower precuneus activity than gender labeling. These findings align with prior reports that affect labeling is more effective at reducing negative emotional response than non-affective labeling [14, 18]. Together these results suggest that salient labels, such as affective or drug-related labels, may be particularly effective in downregulating internal states such as negative affect or craving.

Consistent with our hypothesis and with prior research on affect labeling [43], cue labeling reduced activity in the precuneus, a brain region implicated in cigarette-cue reactivity and craving [38–40, 69, 70], and a region whose activity may have mediated the effect of labeling on craving in the current study. The precuneus and PCC form a DMN core hub that has been associated with both cigarette craving and negative affective states [27] and been identified as a potential therapeutic target for both addiction and internalizing disorders [71]. Indeed, cigarette smoking and internalizing disorders are often comorbid [72], and DMN hyperactivity has been associated with both [73–75]. Activation in this posterior DMN hub specifically has been linked to lower psychological resilience, a measure that includes depression, anxiety, and nicotine use [76]. Thus, labeling may serve as a transdiagnostic intervention for reducing both negative affect and craving, potentially addressing two key psychological triggers of smoking [3, 4, 77]. Future studies are needed to test whether cue labels or affective labels can concurrently modulate both negative affect and craving.

Contrary to our hypothesis, we did not find group-level reductions in other craving-related regions. However, precuneus activity during cue labeling showed positive functional connectivity with several regions previously linked to craving, including the ACC, PCC, and orbitofrontal cortex [40, 54]. Together with the positive correlation of precuneus activity with self-reported craving during cue labeling, these findings strengthen the interpretation that the observed precuneus activity is involved in craving. These results align with prior research demonstrating that labeling is a feasible and effective strategy in clinical populations [19, 23, 78], and they extend that work by demonstrating that labeling can also reduce *craving*.

Labeling is not unique in reducing medial parietal activity. A mindfulness-based intervention for smoking has also reduced cue-elicited activation in this region [7]; interestingly, this intervention involved helping users “identify triggers”, a technique that may implicitly engage cue labeling. Although reappraisal does not typically reduce activation of PCC or precuneus [79], one study found that affect labeling and reappraisal reduced activation in an overlapping region of the precuneus [43]. Overall, these results identify medial parietal cortex as a potential therapeutic target [7, 71, 76] (e.g., for neuromodulation), to be tested in future studies.

Age was the only individual factor that moderated the effects of cue labeling on craving and precuneus activity. Several potential reasons may explain why cue labeling was more effective in older participants. Older adults often report fewer negative emotions and may sometimes exhibit enhanced emotion regulation [reviewed in [48, 80]]. Given the parallels between negative affect and craving [27, 52] and between different types of self-regulation [30, 81], the older group’s better craving regulation may stem from shared mechanisms underlying the regulation of craving and negative affect. Alternatively, older participants may have more experience with managing cravings or greater motivation to quit smoking compared to young adults [1] potentially due to health concerns. However, given that all participants in the current study expressed a strong desire to quit, motivation is not a likely driver of the age effects. Differences in sex distribution and FTND scores between age groups are also unlikely to explain the findings, as neither variable moderated cue labeling effects. Similarly, while age correlates with exposure to smoking, pack-years did not moderate cue labeling effects.

Smoking cessation success rates decline after age 45 [1], highlighting the need for accessible interventions for late midlife and older adults. We found that among participants over 46, cue labeling produced a clinically noticeable and valuable effect [82] (*g* = 0.29), returning cue-induced craving to baseline levels. Given that cue labeling is convenient, essentially cost-free, and devoid of adverse effects, it offers a strong cost-benefit profile compared to other interventions, such as cognitive reappraisal, which require training and cognitive effort. This is especially relevant for older adults, who often have lower cognitive reserve [12]. Age-related cognitive decline may limit the effectiveness of some emotion-regulation strategies, such as reappraisal [49, 50], whereas affect labeling is less cognitively demanding than cognitive reappraisal [83]. Thus, when cognitive reserve is reduced, such as in older adulthood or nicotine withdrawal [11, 84], cue labeling may be a more feasible, acceptable, and effective alternative. However, clinical studies directly comparing these techniques are needed to test these hypotheses.

### Limitations

A limitation of the current study is the relatively small sample size, particularly within each age group. Caution is warranted when generalizing the findings to “older adults”, especially because participants older than 65 were excluded. Another limitation is the specificity of the cue labeling effect, as craving during cue labeling and gender labeling did not differ when considering the whole sample. However, cue labeling showed a larger effect (*g* = 0.29) than gender labeling (*g* = 0.16) (versus cue matching) in the older group, warranting further investigation.

The current design of the cue labeling task also has limitations. Without a craving baseline condition, we cannot determine if cue labeling decreased craving or if cue matching increased it. In affect labeling, when a passive observation baseline is included, affect matching does not alter emotional response, whereas affect labeling reduces it relative to both passive observation and affect matching [14]. Additionally, the current study assessed only the immediate effects of labeling; future work should examine whether these effects persist over time, as observed with affect labeling [18], and whether they extend to changes in smoking behavior.

Other limitations relate to the highly-controlled laboratory design of the study. Whereas the current cue labeling task used forced-choice word selection, other interventions (e.g., mindfulness, reappraisal) typically require participants to self-generate a label or appraisal [7, 30]. In clinical settings, affect labeling can be effective whether the labels are selected [20] or self-generated [19], but their relative efficacy remains unknown. Finally, clinical trials are needed to evaluate the efficacy of cue labeling in real-world contexts, including treatment settings.

### Conclusions

Cue labeling, a scalable and practically zero-cost technique that is easy to use even for people with low cognitive reserve, may be a promising tool for reducing craving, particularly in older individuals. It can easily be used as an adjunct to standard treatments, including pharmacotherapy and counseling. Future studies should test these implications and further examine individual differences in large randomized controlled trials.

## Funding

This work was supported by NIDA grant R21 DA059798 (GT); grants from the Pittsburgh Foundation Charles and Nancy Emmerling Fund, the Pittsburgh Mind Body Center, and the Pennsylvania Department of Health’s Commonwealth Universal Research Enhancement Program (HAT); as well as Mind and Life Institute Varela Awards (HAT and CW); the Pittsburgh Life Sciences Greenhouse Opportunity Fund (GT and JDC), and grant UM1 TR004927 from NCRR and the National Center for Advancing Translational Sciences (UC Irvine). GT was supported by NIMH training grant T32 MH17140, and HAT was supported by NIH training grants KL2 000146, KL2 RR024154-05, and the William Anderson Spickard Jr., MD Endowed Chair in Medicine. Support was also provided by endowments from the Thomas P and Katherine K Pike Chair in Addiction Studies and the Marjorie Greene Family Trust (EDL). The views presented in this article do not necessarily represent those of the funding agencies.

## Supplementary information

Supplemental Material

## Data Availability

The self-report and summary fMRI data presented in this manuscript, as well as the statistical analysis code, are publicly available from the Open Science Framework web site under project title, “Cue Labeling as a Tool to Regulate Craving from Smoking” (https://osf.io/92e4t/).
