# Correction: Cue labeling reduces cigarette craving and associated neural activity

**DOI:** 10.1038/s41386-026-02488-x

**Published:** 2026-07-08

**Authors:** Golnaz Tabibnia, Dara G. Ghahremani, Hilary A. Tindle, J. David Creswell, Cecilia Westbrook, Thomas E. Kraynak, Erica Julson, Edythe D. London

**Affiliations:** 1 Department of Psychology, University of California, Irvine, CA USA; 2 Department of Psychiatry and Biobehavioral Sciences, Semel Institute for Neuroscience and Human Behavior, University of California, Los Angeles, CA USA; 3 Department of Medicine, Vanderbilt University Medical Center, Nashville, TN USA; 4 Geriatric Research Education and Clinical Center for Healthy Longevity, Veterans Affairs Tennessee Valley Healthcare System, Nashville, TN USA; 5 Department of Psychology, Carnegie Mellon University, Pittsburgh, PA USA; 6 Department of Psychiatry, University of Pittsburgh, Pittsburgh, PA USA

**Keywords:** Translational research, Human behaviour

Correction to: *Neuropsychopharmacology* 10.1038/s41386-025-02297-8, published online 17 December 2025

In the final words of the penultimate sentence of the abstract, the phrase “than cue matching” should be corrected to “than gender labeling,” and the corresponding p-value should be updated from “0.01” to “0.001.” The corrected sentence ending should read: “...activity in this region was lower during cue labeling than gender labeling (*Z* = −3.9, *p*-corrected<0.001)”.

The manuscript does not contain text anywhere else that refers to these errors and that would need to be revised.

The original article has been corrected.

