# Supplementary material for: Cue labeling reduces cigarette craving and associated neural activity
Source: Neuropsychopharmacology. 2025 Dec 17;51(5):822–30. doi: 10.1038/s41386-025-02297-8 (PMC13013906; doi:10.1038/s41386-025-02297-8)

SUPPLEMENTAL MATERIAL

SUPPLEMENTARY METHODS

Additional Self-Report Measures

In addition to our focus on the Fagerström Test for Nicotine Dependence (FTND), we collected measures of the following:

- Perceived stress: 10-item Perceived Stress Scale (PSS) (Cohen, Kamarck, and Merm, 1983)
- Depression symptoms: 21-item Beck Depression Inventory (BDI-II) (Beck, Steer, Ball, and Ranieri, 1996)
- Trait anxiety: 20-item State-Trait Anxiety Inventory (STAI) (Spielberger et al., 1983)

The demographic survey included an item on education, with the following categories:

- Some high school or less
- High school graduate or GED certified
- Technical school or some college
- College graduate or more

Stimuli in the Cue Labeling Task

We used 24 neutral images (valence 4-6; arousal <3) from the International Affective Picture System (IAPS) (Lang, Bradley, and Cuthbert, 1997). These images depicted ordinary objects, buildings, and people. Valence and arousal scores were balanced across conditions so that no one condition had more distressing or more arousing stimuli than another. Smoking stimuli (depicting cigarettes, lighters, matches, and/or ashtrays, with or without people) came from the International Smoking Image Series (ISIS) (Gilbert and Rabinovich, 1999) or were purchased from istockphoto.com. For the latter, a sample of people who smoke viewed the images and rated their craving for each image on a 7-point scale; these ratings were averaged and combined with the ratings provided with the ISIS stimuli. We selected 72 images with ratings >2.5 on this 7-point scale. Images were balanced across conditions for average craving ratings.

We generated 4 word-pairs (LIGHTER-ASHTRAY, MATCHES-TOBACCO, SMOKE-MOUTH, and PUFF-PACK) and assigned them to smoking images according to the following criteria: 1) Each word appeared on the left in half the trials and on the right in the other half; and 2) The correct word was on the left in half the trials and on the right in the other half.

fMRI Preprocessing

Results included in this manuscript come from preprocessing performed using *fMRIPrep* 23.1.4 (Esteban et al. (2019); Esteban et al. (2018);

RRID:SCR_016216), which is based on *Nipype* 1.8.6 (K. Gorgolewski et al. (2011); K. J. Gorgolewski et al. (2018); RRID:SCR_002502).

Anatomical data preprocessing

A total of 1 T1-weighted (T1w) images were found within the input BIDS dataset. The T1-weighted (T1w) image was corrected for intensity non-uniformity (INU) with N4BiasFieldCorrection (Tustison et al. 2010), distributed with ANTs (version unknown) (Avants et al. 2008, RRID:SCR_004757), and used as T1w-reference throughout the workflow. The T1w-reference was then skull-stripped with a *Nipype* implementation of theantsBrainExtraction.sh workflow (from ANTs), using OASIS30ANTs as target template. Brain tissue segmentation of cerebrospinal fluid (CSF), white-matter (WM) and gray-matter (GM) was performed on the brain-extracted T1w using fast (FSL (version unknown), RRID:SCR_002823, Zhang, Brady, and Smith 2001). Brain surfaces were reconstructed using recon-all (FreeSurfer 7.3.2, RRID:SCR_001847, Dale, Fischl, and Sereno 1999), and the brain mask estimated previously was refined with a custom variation of the method to reconcile ANTs-derived and FreeSurfer-derived segmentations of the cortical gray-matter of Mindboggle (RRID:SCR_002438, Klein et al. 2017). Volume-based spatial normalization to one standard space (MNI152NLin2009cAsym) was performed through nonlinear registration with antsRegistration (ANTs (version unknown)), using brain-extracted versions of both T1w reference and the T1w template. The following template was were selected for spatial normalization and accessed with *TemplateFlow* (23.0.0, Ciric et al. 2022): *ICBM 152 Nonlinear Asymmetrical template version 2009c* [Fonov et al. (2009), RRID:SCR_008796; TemplateFlow ID: MNI152NLin2009cAsym].

Functional data preprocessing

For each of the 2 BOLD runs found per subject (across all tasks and sessions), the following preprocessing was performed. First, a reference volume and its skull-stripped version were generated using a custom methodology of *fMRIPrep*. Head-motion parameters with respect to the BOLD reference (transformation matrices, and six corresponding rotation and translation parameters) are estimated before any spatiotemporal filtering using mcflirt (FSL , Jenkinson et al. 2002). BOLD runs were slice-time corrected to 0.971s (0.5 of slice acquisition range 0s-1.94s) using 3dTshift from AFNI (Cox and Hyde 1997, RRID:SCR_005927). The BOLD time-series (including slice-timing correction when applied) were resampled onto their original, native space by applying the transforms to correct for head-motion. These resampled BOLD time-series will be referred to as *preprocessed BOLD in original space*, or just *preprocessed BOLD*. The BOLD reference was then co-registered to the T1w reference using bbregister (FreeSurfer) which implements boundary-based registration (Greve and Fischl 2009). Co-registration was configured with six degrees of

freedom. Several confounding time-series were calculated based on the *preprocessed BOLD*: framewise displacement (FD), DVARS and three region-wise global signals. FD was computed using two formulations following Power (absolute sum of relative motions, Power et al. (2014)) and Jenkinson (relative root mean square displacement between affines, Jenkinson et al. (2002)). FD and DVARS are calculated for each functional run, both using their implementations in *Nipype* (following the definitions by Power et al. 2014). The three global signals are extracted within the CSF, the WM, and the whole-brain masks. Additionally, a set of physiological regressors were extracted to allow for component-based noise correction (*CompCor*, Behzadi et al. 2007). Principal components are estimated after high-pass filtering the *preprocessed BOLD* time-series (using a discrete cosine filter with 128s cut-off) for the two *CompCor* variants: temporal (tCompCor) and anatomical (aCompCor). tCompCor components are then calculated from the top 2% variable voxels within the brain mask. For aCompCor, three probabilistic masks (CSF, WM and combined CSF+WM) are generated in anatomical space. The implementation differs from that of Behzadi et al. in that instead of eroding the masks by 2 pixels on BOLD space, a mask of pixels that likely contain a volume fraction of GM is subtracted from the aCompCor masks. This mask is obtained by dilating a GM mask extracted from the FreeSurfer's *aseg* segmentation, and it ensures components are not extracted from voxels containing a minimal fraction of GM. Finally, these masks are resampled into BOLD space and binarized by thresholding at 0.99 (as in the original implementation). Components are also calculated separately within the WM and CSF masks. For each CompCor decomposition, the *k* components with the largest singular values are retained, such that the retained components' time series are sufficient to explain 50 percent of variance across the nuisance mask (CSF, WM, combined, or temporal). The remaining components are dropped from consideration. The head-motion estimates calculated in the correction step were also placed within the corresponding confounds file. The confound time series derived from head motion estimates and global signals were expanded with the inclusion of temporal derivatives and quadratic terms for each (Satterthwaite et al. 2013). Frames that exceeded a threshold of 0.5 mm FD or 1.5 standardized DVARS were annotated as motion outliers. Additional nuisance timeseries are calculated by means of principal components analysis of the signal found within a thin band (*crown*) of voxels around the edge of the brain, as proposed by (Patriat, Reynolds, and Birn 2017). The BOLD time-series were resampled into standard space, generating a *preprocessed BOLD run in MNI152NLin2009cAsym space*. First, a reference volume and its skull-stripped version were generated using a custom methodology of *fMRIPrep*. All resamplings can be performed with *a single interpolation step* by composing all the pertinent transformations (i.e. head-motion transform matrices, susceptibility distortion correction when available, and co-registrations to anatomical and output spaces). Gridded (volumetric) resamplings were performed usingantsApplyTransforms (ANTs), configured with

Lanczos interpolation to minimize the smoothing effects of other kernels (Lanczos 1964). Non-gridded (surface) resamplings were performed using mri_vol2surf(FreeSurfer).

Many internal operations of *fMRIPrep* use *Nilearn* 0.10.1 (Abraham et al. 2014, RRID:SCR_001362), mostly within the functional processing workflow. For more details of the pipeline, see the section corresponding to workflows in *fMRIPrep*'s documentation.

Sensitivity (Outlier) Analysis of the Precuneus-Craving Association

Using standardized residuals with absolute values > 3, we identified one extreme outlier. To identify additional influential points, we used Cook's distance, a measure of the influence of individual observations on the overall regression model. Observations with Cook's distance values exceeding the threshold of 4/n (where n=42) were classified as influential points. This criterion identified 3 cases (which included the one extreme outlier) that had a disproportionate impact on the regression coefficient and model fit. In subsequent sensitivity analyses, the extreme outlier or all 3 influential points were removed from the linear regression model of precuneus activation on craving during cue labeling.

Mediation Analysis

To test whether the effect of cue labeling on craving can be explained by the impact of cue labeling on precuneus activity, we conducted a mediation analysis, using the *mediation* package in R (Tingley et al., 2014). Specifically, we used a Quasi-Bayesian 3-variable model with 500,000 simulations. In this model, X was condition (cue labeling vs. cue matching), Y was self-reported craving, and M was activity in the precuneus cluster identified in the contrast of cue labeling versus cue matching. The covariates of age, sex, FTND, and meanFD were used in both LMM models (for path a and path c'). This analysis was conducted on the subset of 42 participants who had both usable fMRI and self-reported craving data.

SUPPLEMENTARY RESULTS

Moderation Analyses

In *post hoc* moderation analyses (that included a single interaction term), none of the following demographic or clinical variables moderated the effect of cue labeling vs. cue matching on craving.

- Perceived Stress (PSS): $F_{1,44}$=2.1, p=0.16
- Depression (BDI): $F_{1,44}$=0.01, p=0.92
- Trait Anxiety (STAI): $F_{1,44}$=0.01, p=0.92
- Years of education: $F_{1,43}$=0.34, p=0.57
- Annual Income: $F_{1,44}$=0.14, p=0.71

Although perceived stress did not significantly moderate the effect of labeling on craving, there was a trend toward an increasing difference between conditions with increasing stress.

None of these demographic or clinical variables moderated the effect of cue labeling on brain activation (in models that included age, sex, and mFD as additional covariates), except for years of education. Increasing years of education was associated with greater activation in supramarginal gyrus / postcentral gyrus during cue labeling compared to cue matching (x=-23, y=-30, z=37; Z= 3.5, k=68).

Sensitivity (Outlier) Analysis of the Precuneus-Craving Association

After removing the one extreme outlier, results of the linear regression model of precuneus activation on craving during cue labeling remained significant (β=0.506, p=0.003; Fig. S7A). Results were still significant after removing all 3 influential points (β=0.458, p=0.012; Fig. S7B).

Mediation Analysis

In a *post hoc* mediation analysis (N=42; Fig. S4), the total effect of labeling on craving (path c) did not quite reach significance (β=-0.093, 95% confidence interval or CI=[-0.190, 0.004], p=0.061) in this smaller sample. Nonetheless, this sub-threshold effect was significantly mediated by activity in the precuneus (β=-0.096, CI=[-0.200, -0.002], p=0.045). After controlling for activity in this precuneus region, the remaining path from cue labeling to craving (path c', the direct effect) was not significant (β=0.003, CI=[-0.130, 0.136], p=0.959). This mediation analysis was likely underpowered, as indicated by a significant mediation effect and a non-significant total effect. However, given that in the larger sample of participants who provided self-reported craving (N=46), there was a significant total effect of labeling on craving ($F_{(1, 45)}$=-4.1, p=0.049, as reported in the main manuscript), we interpret the results of the mediation analysis as an indication that the effect of labeling on craving was likely due in large part to the impact of labeling on precuneus activity. However, a larger sample size is needed to confirm this conclusion.

Craving in Older vs. Younger Groups

In a *post hoc* comparison, self-reported craving in the older group during cue matching (M=2.85, SD=0.66) did not significantly differ (t_{44}=1.26, p=0.22) from that in the younger group (M=3.17, SD=1.05). The difference in craving during cue labeling in

the older group (M=2.65, SD=0.70) versus the younger group (M=3.18, SD=1.12) was marginally significant (t_{44}=1.91, p=0.06).

References

Abraham, Alexandre, Fabian Pedregosa, Michael Eickenberg, Philippe Gervais, Andreas Mueller, Jean Kossaifi, Alexandre Gramfort, Bertrand Thirion, and Gael Varoquaux. 2014. "Machine Learning for Neuroimaging with Scikit-Learn." *Frontiers in Neuroinformatics* 8. https://doi.org/10.3389/fninf.2014.00014.

Avants, B. B., C. L. Epstein, M. Grossman, and J. C. Gee. 2008. "Symmetric Diffeomorphic Image Registration with Cross-Correlation: Evaluating Automated Labeling of Elderly and Neurodegenerative Brain." *Medical Image Analysis* 12 (1): 26–41. https://doi.org/10.1016/j.media.2007.06.004.

Beck, A. T., Steer, R. A., Ball, R., & Ranieri, W. F. 1996. Comparison of Beck Depression Inventories-IA and-II in psychiatric outpatients. *Journal of personality assessment*, *67*(3), 588-597.

Behzadi, Yashar, Khaled Restom, Joy Liau, and Thomas T. Liu. 2007. "A Component Based Noise Correction Method (CompCor) for BOLD and Perfusion Based fMRI." *NeuroImage* 37 (1): 90–101. https://doi.org/10.1016/j.neuroimage.2007.04.042.

Ciric, R., William H. Thompson, R. Lorenz, M. Goncalves, E. MacNicol, C. J. Markiewicz, Y. O. Halchenko, et al. 2022. "TemplateFlow: FAIR-Sharing of Multi-Scale, Multi-Species Brain Models." *Nature Methods* 19: 1568–71. https://doi.org/10.1038/s41592-022-01681-2.

Cohen S, Kamarck T, Mermelstein R. 1983. A global measure of perceived stress, journal of health and social behavior, vol. 24.

Cox, Robert W., and James S. Hyde. 1997. "Software Tools for Analysis and Visualization of fMRI Data." *NMR in Biomedicine* 10 (4-5): 171–78. https://doi.org/10.1002/(SICI)1099-1492(199706/08)10:4/5<171::AID-NBM453>3.0.CO;2-L.

Dale, Anders M., Bruce Fischl, and Martin I. Sereno. 1999. "Cortical Surface-Based Analysis: I. Segmentation and Surface Reconstruction." *NeuroImage* 9 (2): 179–94. https://doi.org/10.1006/nimg.1998.0395.

Esteban, Oscar, Ross Blair, Christopher J. Markiewicz, Shoshana L. Berleant, Craig Moodie, Feilong Ma, Ayse Ilkay Isik, et al. 2018. "fMRIPrep 23.1.4." *Software*. https://doi.org/10.5281/zenodo.852659.

Esteban, Oscar, Christopher Markiewicz, Ross W Blair, Craig Moodie, Ayse Ilkay Isik, Asier Erramuzpe Aliaga, James Kent, et al. 2019. "fMRIPrep: A Robust Preprocessing Pipeline for Functional MRI." *Nature Methods* 16: 111–16. https://doi.org/10.1038/s41592-018-0235-4.

Fonov, VS, AC Evans, RC McKinstry, CR Almli, and DL Collins. 2009. "Unbiased Nonlinear Average Age-Appropriate Brain Templates from Birth to

Adulthood." *NeuroImage* 47, Supplement 1: S102.https://doi.org/10.1016/S1053-8119(09)70884-5.

Gilbert, D. G., & Rabinovich, N. E. (1999). International smoking image series (with neutral counterparts), version 1.2. *Carbondale, Integrative Neuroscience Laboratory, Department of Psychology, Southern Illinois University*.

Gorgolewski, K., C. D. Burns, C. Madison, D. Clark, Y. O. Halchenko, M. L. Waskom, and S. Ghosh. 2011. "Nipype: A Flexible, Lightweight and Extensible Neuroimaging Data Processing Framework in Python."*Frontiers in Neuroinformatics* 5: 13. https://doi.org/10.3389/fninf.2011.00013.

Gorgolewski, Krzysztof J., Oscar Esteban, Christopher J. Markiewicz, Erik Ziegler, David Gage Ellis, Michael Philipp Notter, Dorota Jarecka, et al. 2018. "Nipype." *Software*. https://doi.org/10.5281/zenodo.596855.

Greve, Douglas N, and Bruce Fischl. 2009. "Accurate and Robust Brain Image Alignment Using Boundary-Based Registration." *NeuroImage* 48 (1): 63–72. https://doi.org/10.1016/j.neuroimage.2009.06.060.

Jenkinson, Mark, Peter Bannister, Michael Brady, and Stephen Smith. 2002. "Improved Optimization for the Robust and Accurate Linear Registration and Motion Correction of Brain Images." *NeuroImage* 17 (2): 825–41. https://doi.org/10.1006/nimg.2002.1132.

Klein, Arno, Satrajit S. Ghosh, Forrest S. Bao, Joachim Giard, Yrjö Häme, Eliezer Stavsky, Noah Lee, et al. 2017. "Mindboggling Morphometry of Human Brains." *PLOS Computational Biology* 13 (2): e1005350. https://doi.org/10.1371/journal.pcbi.1005350.

Lanczos, C. 1964. "Evaluation of Noisy Data." *Journal of the Society for Industrial and Applied Mathematics Series B Numerical Analysis* 1 (1): 76–85. https://doi.org/10.1137/0701007.

Lang, P. J., Bradley, M. M., & Cuthbert, B. N. (1997). International affective picture system (IAPS): Technical manual and affective ratings. *NIMH Center for the Study of Emotion and Attention*, *1*(39-58), 3.

Patriat, Rémi, Richard C. Reynolds, and Rasmus M. Birn. 2017. "An Improved Model of Motion-Related Signal Changes in fMRI." *NeuroImage* 144, Part A (January): 74–82. https://doi.org/10.1016/j.neuroimage.2016.08.051.

Power, Jonathan D., Anish Mitra, Timothy O. Laumann, Abraham Z. Snyder, Bradley L. Schlaggar, and Steven E. Petersen. 2014. "Methods to Detect, Characterize, and Remove Motion Artifact in Resting State fMRI."*NeuroImage* 84 (Supplement C): 320–41. https://doi.org/10.1016/j.neuroimage.2013.08.048.

Satterthwaite, Theodore D., Mark A. Elliott, Raphael T. Gerraty, Kosha Ruparel, James Loughead, Monica E. Calkins, Simon B. Eickhoff, et al. 2013. "An improved framework for confound regression and filtering for control of motion artifact in the preprocessing

of resting-state functional connectivity data.” *NeuroImage* 64 (1): 240–56. https://doi.org/10.1016/j.neuroimage.2012.08.052.

Speilberger, C. D., Gorsuch, R., Lushene, R., Vagg, P. R., & Jacobs, G. A. (1983). Manual for the state-trait anxiety inventory. *Palo Alto, CA: Consulting Psychologists*.

Tingley, D., Yamamoto, T., Hirose, K., Imai, K. and Keele, L. (2014). "mediation: R package for Causal Mediation Analysis", *Journal of Statistical Software, Vol. 59*, No. 5, pp. 1-38.

Tustison, N. J., B. B. Avants, P. A. Cook, Y. Zheng, A. Egan, P. A. Yushkevich, and J. C. Gee. 2010. “N4ITK: Improved N3 Bias Correction.” *IEEE Transactions on Medical Imaging* 29 (6): 1310–20. https://doi.org/10.1109/TMI.2010.2046908.

Zhang, Y., M. Brady, and S. Smith. 2001. “Segmentation of Brain MR Images Through a Hidden Markov Random Field Model and the Expectation-Maximization Algorithm.” *IEEE Transactions on Medical Imaging* 20 (1): 45–57. https://doi.org/10.1109/42.906424.

Table S1. Brain activation during matching versus labeling of smoking cues (N=42), excluding the 8 participants without craving ratings.

Region[a]	Cluster size (voxels)	*Z* (max)[b]	X[c]	Y[c]	Z[c]
Cue Matching - Cue Labeling					
Occipital pole / Lateral Occipital Cortex	338	6.32	31.6	-90.9	17.5
Lateral occipital / Precuneus	155	4.75	-19.6	-65.2	46.3
Temporal Occipital Fusiform Cortex	77	4.59	28.4	-49.2	-14.5
Precuneus	53	4.55	-3.61	-68.4	49.5
Occipital Fusiform / T.O. Fusiform Cortex	27	4.34	-29.2	-71.6	-14.5
Cue Matching - Gender Labeling					
Precuneus	170	4.86	-10	-68.4	36.7
Lateral Occipital Cortex	132	5.56	31.6	-81.3	20.7
Temporal Occipital Fusiform	77	5.43	-26	-52.4	-14.5
Temporal Occipital Fusiform	56	5.16	25.2	-49.2	-14.5
Lateral Occipital Cortex	37	4.42	47.6	-68.4	-8.1
Lateral Occipital Cortex	27	4.11	22	-71.6	52.7
Lateral Occipital Cortex	23	4.7	-51.7	-71.6	-4.9
Inferior Temporal Gyrus	21	4.47	-54.9	-46	-17.7

Whole-brain voxel-wise contrasts are presented. All results were cluster corrected at a voxel-height threshold of $p<0.001$ ($Z>3.1$) and a cluster-size threshold of $p<0.05$.

[a] Anatomical labels based on the Harvard-Oxford Structural Atlas

[b] *Z*-statistic of peak voxel

[c] MNI coordinates of peak voxel within cluster

Table S2
Comparison of neural activity between cue labeling and gender labeling.

Region[a]	Cluster size (voxels)	*Z* (max)[b]	X[c]	Y[c]	Z[c]
Gender Labeling - Cue Labeling					
Angular G. / Lateral Occipital	439	5.06	54	-55.6	43.1
MFG	342	4.51	41.2	21.2	33.5
Angular G. / Supramarginal G.	149	4.6	-54.9	-52.4	27.1
Precuneus	74	3.9	2.79	-74.8	49.5
Frontal Pole	64	4.47	38	59.7	-1.7
IFG opercularis	62	3.59	-58.1	18	11.1
SFG / paracingulate	55	3.45	6	37.3	49.5
Cue Labeling – Gender Labeling					
T.O. Fusiform / WM	185	3.89	-45.3	-39.6	-8.1
Caudate / Subcallosal Cortex	142	3.88	12.4	21.2	-8.1
WM	69	3.78	25.2	34.1	14.3
Anterior Cingulate Cortex	58	3.39	-6.82	-7.58	23.9

Whole-brain voxel-wise contrasts are presented. All results were cluster corrected at a voxel-height threshold of $p<0.001$ ($Z>3.1$) and a cluster-size threshold of $p<0.05$.
[a] Anatomical labels based on the Harvard-Oxford Structural Atlas
[b] *Z*-statistic of peak voxel
[c] MNI coordinates of peak voxel within cluster
G. = Gyrus
MFG = Middle Frontal Gyrus
IFG = Inferior Frontal Gyrus
SFG = Superior Frontal Gyrus
T.O. = Temporal Occipital
WM = White Matter

Table S3
Association of precuneus activity with other brain regions during cue labeling

Region[a]	Cluster size (voxels)	*Z* (max)[b]	X[c]	Y[c]	Z[c]
Positive association					
Frontal Operculum, IFG (pars triangularis), OFC	191	4.82	-45.3	27.7	-1.7
Angular G, Lateral Occipital Cortex	153	4.64	57.2	-55.6	36.7
Angular G, Supramarginal G, Lateral Occipital Cortex	144	4.64	-48.5	-55.6	49.5
Middle Frontal G	110	5.22	-38.8	8.4	46.3
Posterior Cingulate Cortex (PCC)	90	4.64	2.8	-30.0	33.5
Paracingulate G, dorsal ACC	80	4.52	6.0	43.7	20.7
IFG (pars opercularis)	72	4.46	44.4	8.4	20.7
Paracingulate G, Frontal Pole	44	4.2	-10.0	46.9	17.5
Dorsal thalamus / caudate	36	4.07	2.8	-7.6	11.1
Negative association					
NA					

Whole-brain voxel-wise connectivity results are presented. All results were cluster corrected at a voxel-height threshold of $p<0.001$ (Z>3.1) and a cluster-size threshold of $p<0.05$.
[a] Anatomical labels based on the Harvard-Oxford Structural Atlas
[b] *Z*-statistic of peak voxel
[c] MNI coordinates of peak voxel within cluster
G. = Gyrus
IFG = Inferior Frontal Gyrus
OFC = Orbitofrontal Cortex

Table S4
Older group's participant characteristics

**Older group (Age≥47)**	fMRI (N=26)	Craving (N=23)
Age [M (SD)]	54.2 (5.4)	53.6 (4.8)
Female	35%	44%
Race		
African-American	42%	52%
Caucasian-American	54%	48%
Other	4%	0%
Annual Household Income		
<$20,000	50%	52%
$20,000-50,000	19%	13%
$50,000-75,000	31%	35%
>$75,000	0%	0%
Beck Depression Inventory II		
Score [M (s.d.)]	6.2 (4.4)	7.4 (6.7)
Scored ≤13	89%	83%
Smoking status [M (s.d.)]		
Nicotine dependence (FTND)	4.6 (2.4)	4.0 (2.3)
Cigarettes per day	18.6 (6.5)	17.4 (6.2)
Packyears	29.1 (13.2)	28.6 (12.3)
Baseline CO level (parts per million)	17.3 (10.0)	16.3 (8.1)
fMRI CO level (parts per million)	5.8 (4.5)	4.9 (3.9)

CO = carbon monoxide
FTND= Fagerström Test for Nicotine Dependence
M = mean
SD = standard deviation

Table S5
Younger group's participant characteristics

**Younger group (Age<47)**	fMRI (N=24)	Craving (N=23)
Age [M (SD)]	37.0 (7.4)	37.2 (7.7)
Female	63%	61%
Race		
African-American	42%	44%
Caucasian-American	54%	52%
Other	4%	4%
Annual Household Income		
<$20,000	58%	52%
$20,000-50,000	21%	26%
$50,000-75,000	13%	13%
>$75,000	8%	9%
Beck Depression Inventory II		
Score [M (s.d.)]	8.0 (6.2)	7.7 (6.4)
Scored ≤13	83%	83%
Smoking status [M (s.d.)]		
Nicotine dependence (FTND)	5.4 (1.9)	5.5 (2.1)
Cigarettes per day	16.0 (5.5)	16.3 (7.5)
Packyears	16.7 (10.1)	18.1 (13.9)
Baseline CO level (parts per million)	14.0 (6.5)	15.0 (6.6)
fMRI CO level (parts per million)	5.6 (4.1)	5.2 (3.8)

CO = carbon monoxide
FTND= Fagerström Test for Nicotine Dependence
M = mean
SD = standard deviation

Fig. S1

Task design. Each block began with a 2.5-s instruction screen indicating “Match” or “Label”, followed by a 0.5-s interstimulus interval. Then participants had 4 s to select one of two responses that matched the image above, followed by an interstimulus interval of jittered duration (average 1 s, range 0–2.5 s, distributed exponentially), before the next trial. After each block of 6 trials, participants had 4 s to rate the strength of their craving on a 5-point scale, followed by a 5-s rest (fixation cross) before the start of the next block.

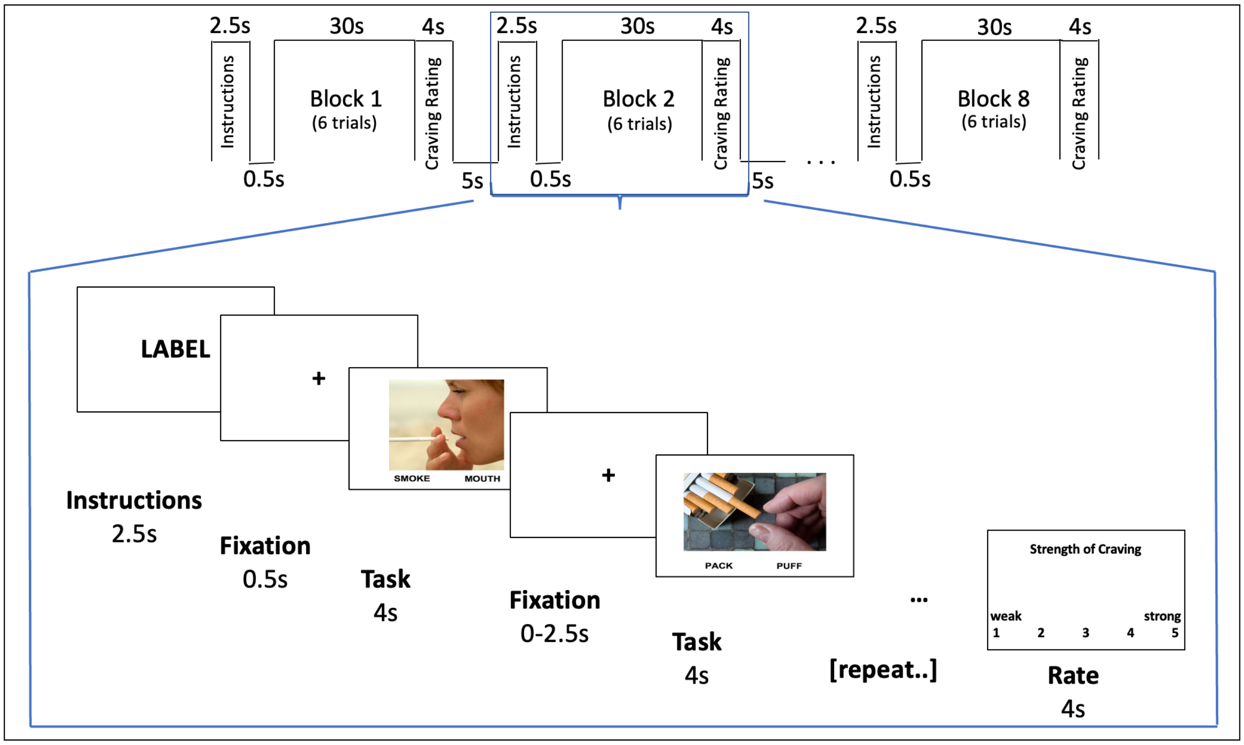

Fig. S2

Neural activation during cue matching versus cue labeling. Thresholded z-stat images (*Z*<3.1, cluster-size threshold of *p*<0.05) show activation in a lateral occipital cluster that includes the precuneus, as well as lingual gyrus and temporal occipital fusiform.

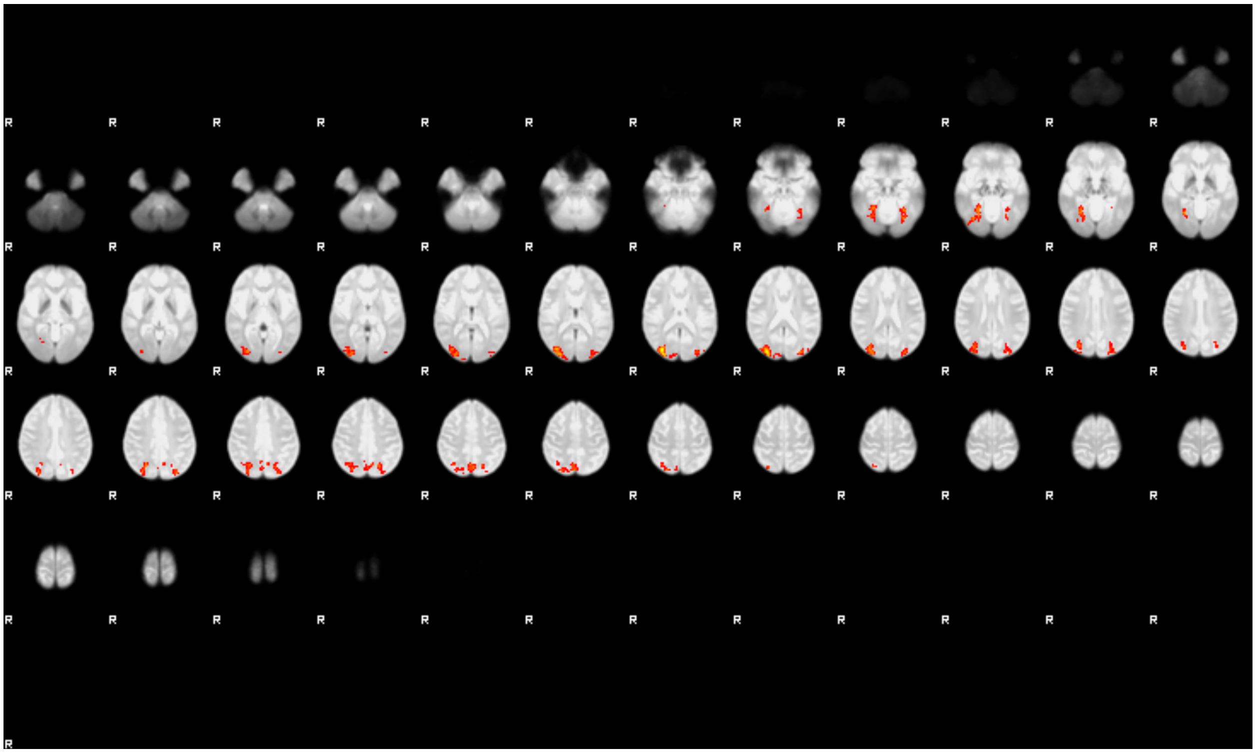

Fig. S3

Neural activation during cue matching versus gender labeling. Thresholded z-stat images (*Z*<3.1, cluster-size threshold of *p*<0.05) show activation in a precuneus cluster, as well as lateral occipital, occipital pole, middle temporal gyrus, lingual gyrus, and temporal occipital fusiform.

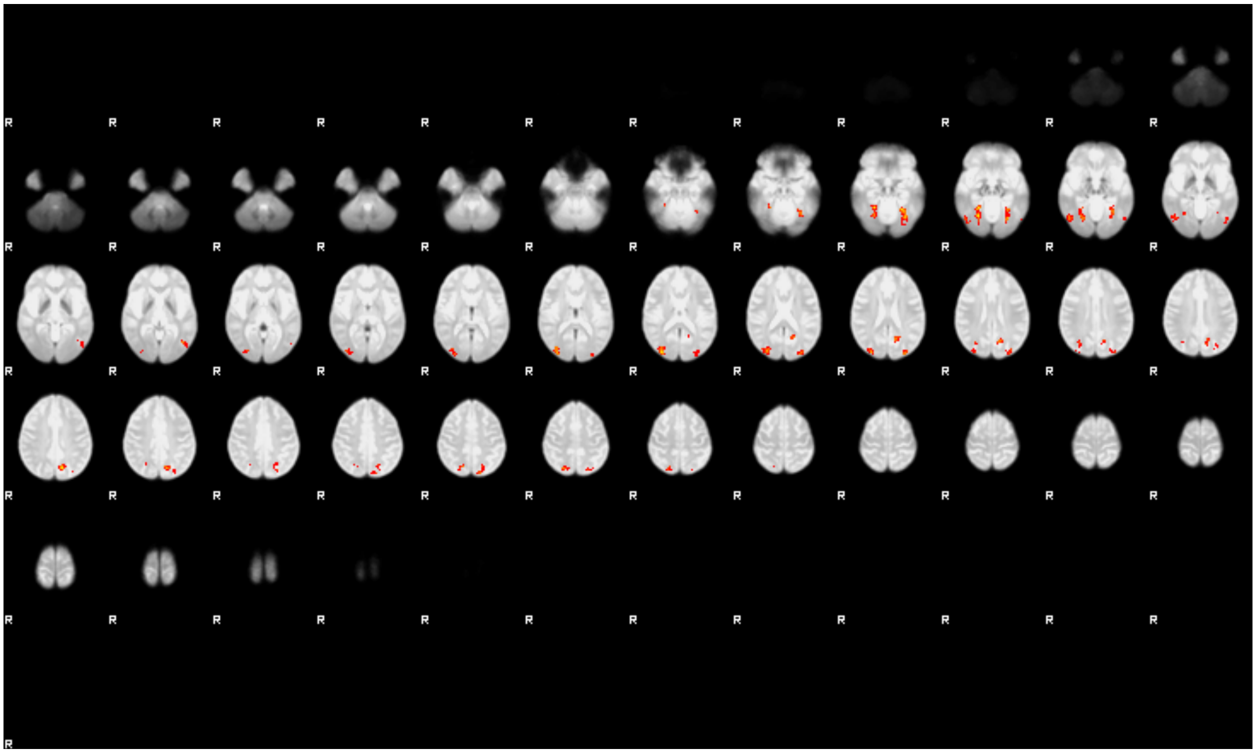

Fig. S4

Exploratory mediation model. Preliminary results suggest that activity in the precuneus fully mediates the effect of labeling on self-reported craving. Standardized path coefficients are presented along with 95% confidence intervals in brackets. Path a refers to the effect of condition (labeling vs. matching) on precuneus activity; path b refers to the association between precuneus activity and craving, controlling for labeling condition; path c' refers to the direct effect of condition on craving, controlling for precuneus activity; and path a*b refers to the indirect effect of labeling on craving, or the mediation effect.
* $p < 0.05$, *** $p < 0.001$

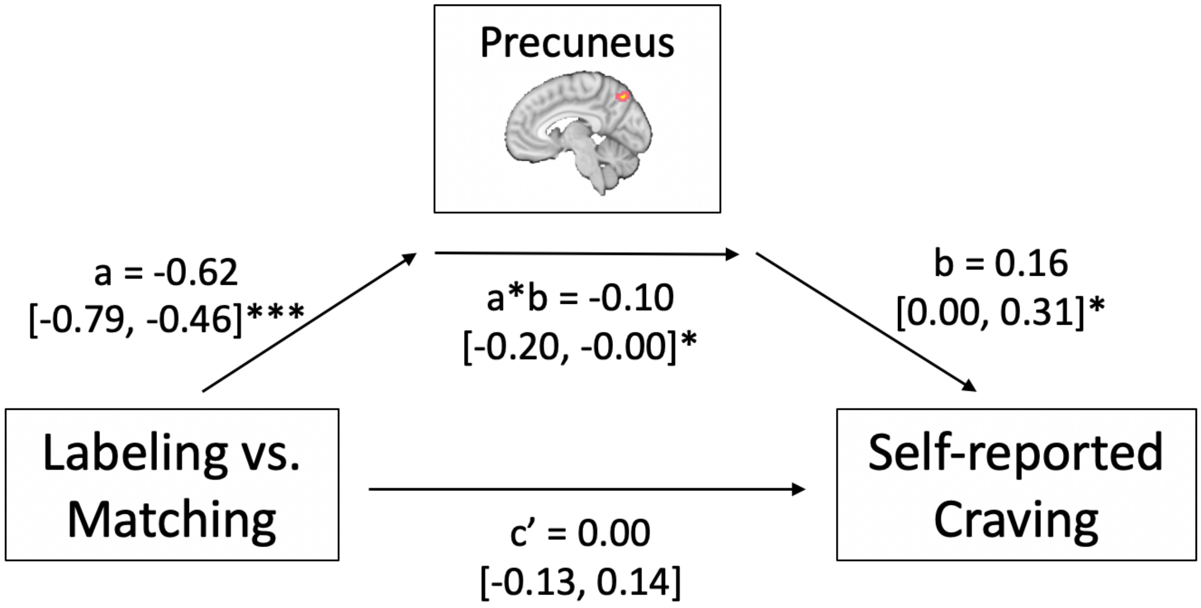

Fig. S5

Functional connectivity of precuneus during cue labeling. Thresholded z-stat images (*Z*<3.1, cluster-size threshold of *p*<0.05) show positive functional connectivity of precuneus with several brain regions, including frontal operculum / orbitofrontal cortex, posterior cingulate cortex, paracingulate gyrus / anterior cingulate cortex, inferior frontal gyrus (pars opercularis), paracingulate gyrus / frontal pole, and dorsal thalamus / caudate.

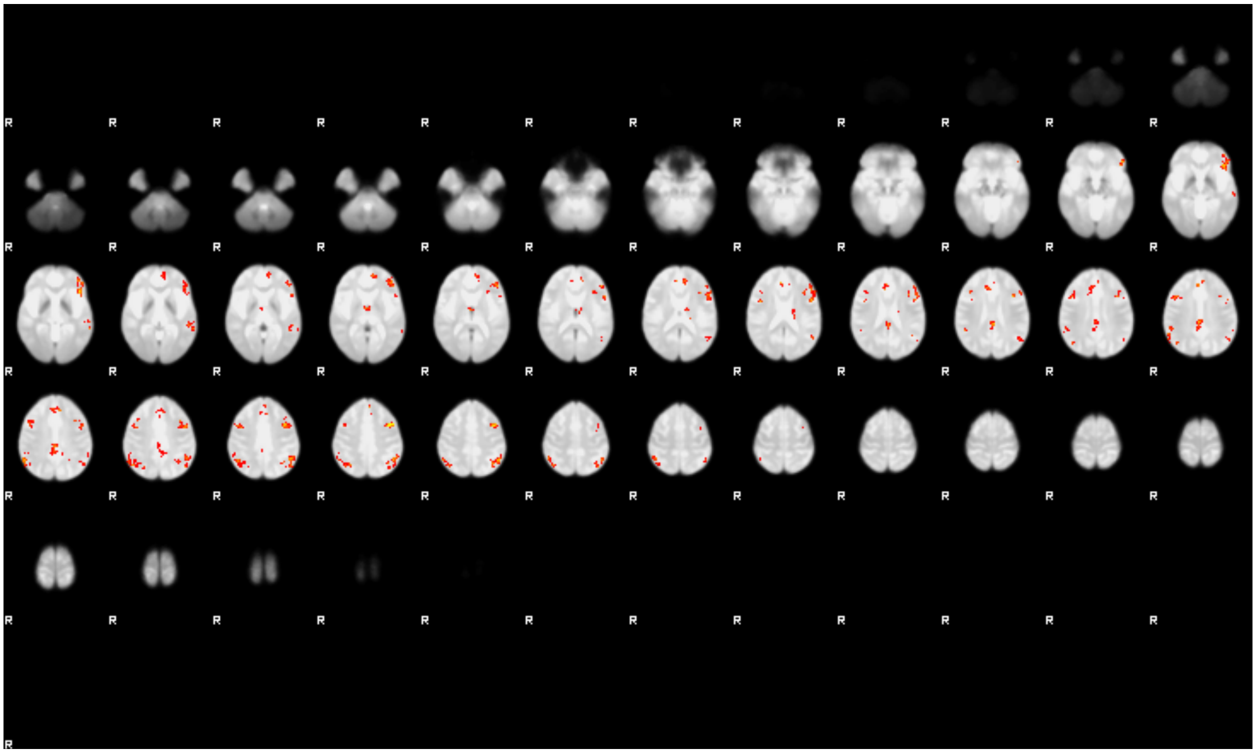

Fig. S6

Craving ratings of the younger group. The younger group's craving ratings during cue labeling (M=3.18, SD=1.12) did not differ ($F_{1,22}$=0.004, p=0.95, Hedges' g=0.003) from their ratings during cue matching (3.17, SD=1.05).

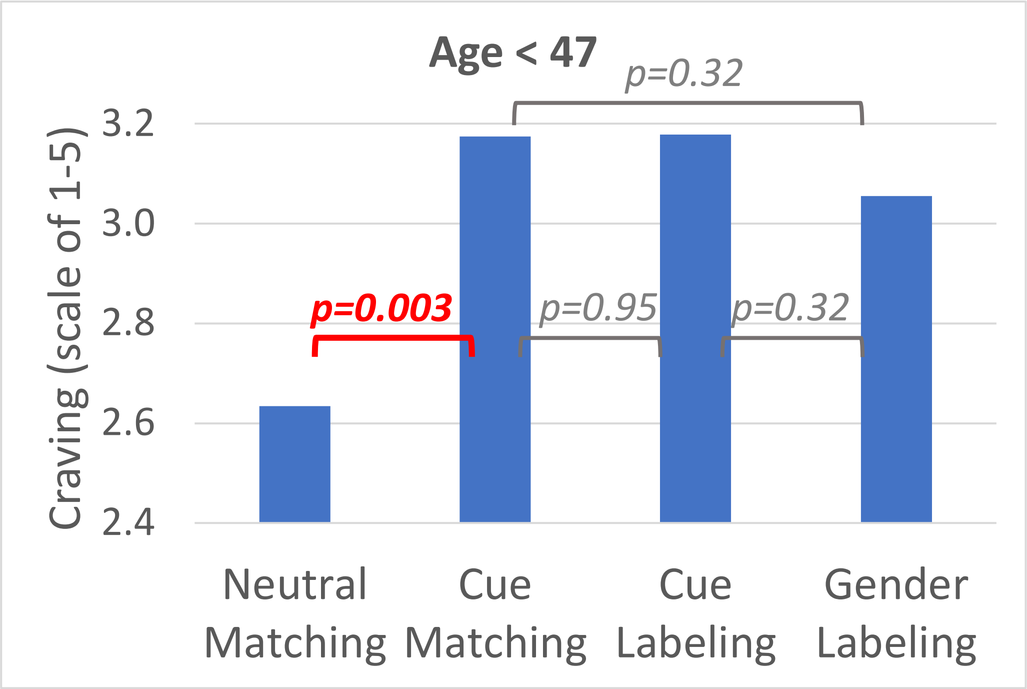

Fig. S7

Sensitivity analyses of the association between craving and precuneus activation. After removing the extreme outlier identified by the standardized residuals criterion of absolute values > 3 (A), or after removing the 3 influential points identified by the Cook's distance criterion of values > 4/N (B), precuneus activity during cue labeling was still positively associated with self-reported craving during those trials. Y-axis represents partial residuals of the fMRI contrast estimates during cue labeling.

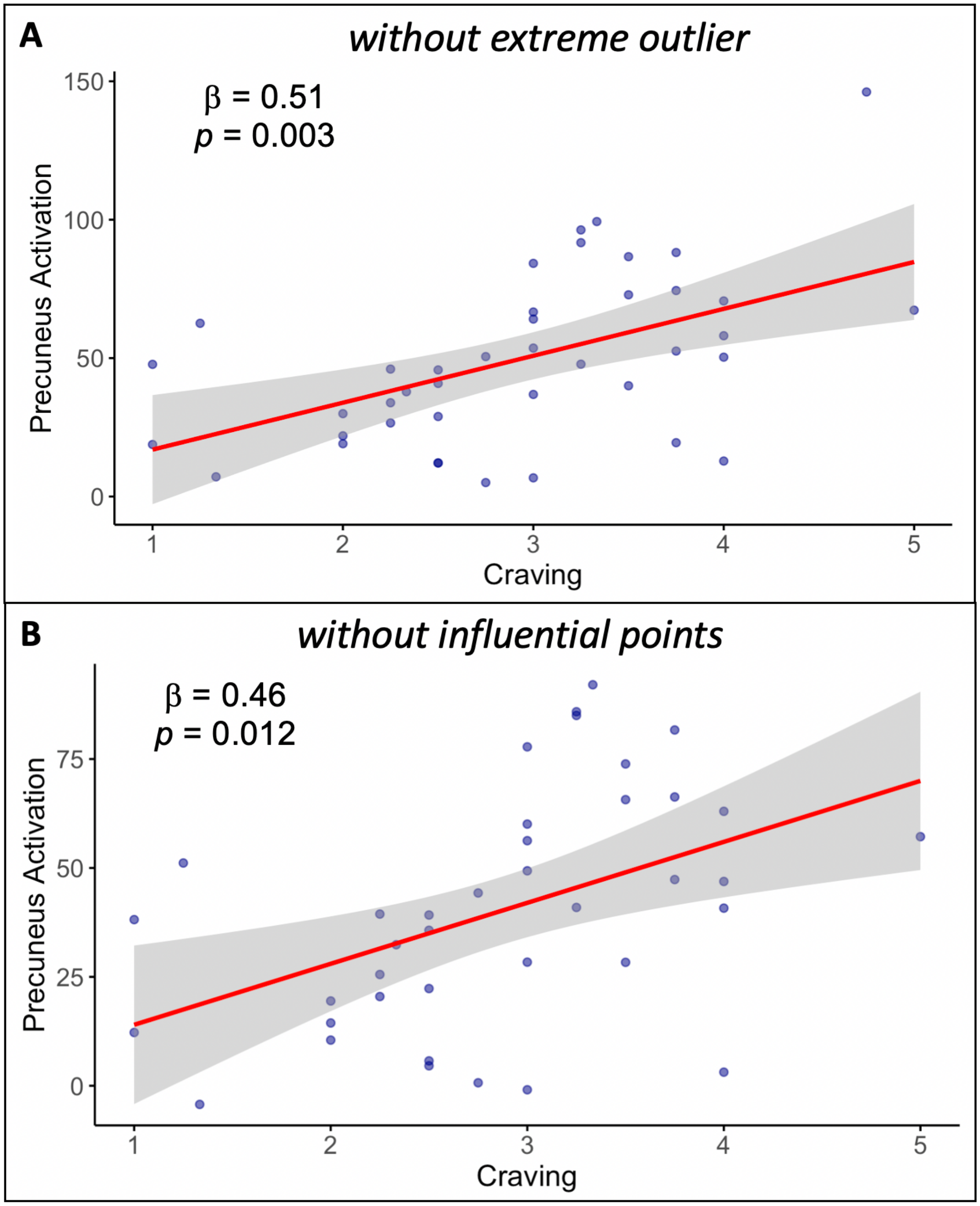

Supplement: Supplementary file 1 — Supplemental Material [file 41386_2025_2297_MOESM1_ESM.pdf]
